# The gene expression of the neuronal protein, SLC38A9, changes in mouse brain after *in vivo* starvation and high-fat diet

**DOI:** 10.1371/journal.pone.0172917

**Published:** 2017-02-24

**Authors:** Sofie V. Hellsten, Mikaela M. Eriksson, Emilia Lekholm, Vasiliki Arapi, Emelie Perland, Robert Fredriksson

**Affiliations:** Department of Pharmaceutical Bioscience, Molecular Neuropharmacology, Uppsala University, Uppsala SE, Sweden; International Nutrition Inc, UNITED STATES

## Abstract

SLC38A9 is characterized as a lysosomal component of the amino acid sensing Ragulator-RAG GTPase complex, controlling the mechanistic target of rapamycin complex 1 (mTORC1). Here, immunohistochemistry was used to map SLC38A9 in mouse brain and staining was detected throughout the brain, in cortex, hypothalamus, thalamus, hippocampus, brainstem and cerebellum. More specifically, immunostaining was found in areas known to be involved in amino acid sensing and signaling pathways e.g. piriform cortex and hypothalamus. SLC38A9 immunoreactivity co-localized with both GABAergic and glutamatergic neurons, but not with astrocytes. SLC38A9 play a key role in the mTORC1 pathway, and therefore we performed *in vivo* starvation and high-fat diet studies, to measure gene expression alterations in specific brain tissues and in larger brain regions. Following starvation, *Slc38a9* was upregulated in brainstem and cortex, and in anterior parts of the brain (Bregma 3.2 to -2.1mm). After high-fat diet, *Slc38a9* was specifically upregulated in hypothalamus, while overall downregulation was noticed throughout the brain (Bregma 3.2 to -8.6mm).

## Introduction

Solute carriers (SLCs) are the largest group of transporters in the human genome [1] and they function as passive transporters, coupled transporters, or exchangers [2]. The transporters are membrane bound and found in the plasma [3], vesicle [4], mitochondrial [5], peroxisomal [6] and lysosomal [7] membranes. The 395 SLCs found so far are divided into 52 families, where the members in each family share at least 20% amino acid sequence identity to another member, and hence functional properties [8]. SLCs are transporters for a variety of substrates, e.g. drugs, ions, amino acids, sugars and nucleotides [9].

The system A and system N sodium-coupled neutral amino acid transporter family, SLC38 family, has 11 members [10]. Today, seven members are functionally characterized and divided into system A, SLC38A1 [11], SLC38A2 [12], SLC38A4 [13] and SLC38A8 [14], or system N, SLC38A3 [15], SLC38A5 [16] and SLC38A7 [17]. System A transport of small neutral amino acids, in particular alanine, serine and glutamine, is sodium coupled [18], while system N transport of mainly glutamine, asparagine and histidine [19] is sodium coupled with hydrogen in exchange [15]. SLC38A9 is not yet characterized into either transport system, but was recently implicated as a lysosomal component of the amino acid sensing Ragulator-RAG GTPase complex, controlling the mechanistic target of rapamycin complex 1 (mTORC1) [20, 21]. Furthermore, SLC38A9 is a low affinity transporter for arginine, glutamine and asparagine [21] and signals mainly arginine sufficiency to mTORC1 [22]. In mammalian cells, there are two major amino acid sensing pathways, the mTORC1 pathway and the amino acid responsive (AAR) pathway [23]. Amino acid transporters play key roles both upstream and downstream in these pathways, since they can sense intracellular and extracellular amino acid concentrations. Both pathways are involved in gene expression regulation of amino acid transporters [24]. mTORC1 function as a signal integrator that receive information about nutrient levels e.g. glucose, amino acids, growth factors and energy, and can therefore regulate the metabolism and cell growth [25, 26]. Amino acids increase the mTORC1 activation on lysosomes via the Rag GTPases and the Ragulator complex, and amino acids thereby control protein synthesis and cell growth [26]. In the brain, SLC38A1 [27, 28] and SLC38A2 [29] are located to neurons and astrocytes. SLC38A3 [30, 31] and SLC38A5 [32] are astrocytic, while SLC38A6 [33], SLC38A7 [17] and SLC38A8 [14] are neuronal. SLC38A9 is ubiquitously expressed in the body, especially in parathyroid gland, thyroid gland, testis and adrenal gland [34].

Here we present histological data of SLC38A9, as well as gene expression data for *Slc38a9* following altered nutrient availability. SLC38A9 immunoreactivity was mapped in mouse brain using non-fluorescent immunohistochemistry with a custom made anti-SLC38A9 antibody. Furthermore, a commercially available anti-SLC38A9 antibody was used to verify the staining in mouse brain. In addition, fluorescent immunohistochemistry was used to determine in which cell types SLC38A9 co-localize with specific cell markers. Moreover, *Slc38a9* gene expression changes in mouse brain after starvation and high-fat diet was studied to gain information about the relation of *Slc38a9* and nutrient availability *in vivo*.

## Material and methods

### Ethical statement

All experiments using C57Bl6/J mice (Taconic M&B, Denmark) were approved by the local ethical committee in Uppsala, in unity with the EU-directive 2010/63, (Permit number: C67/13 and C419/12). Adult male mice were used in all experiments and they were kept in a temperature controlled room on a 12h light-dark cycle, with food and water *ad libitum*, unless otherwise stated. All chemicals used were purchased from Sigma-Aldrich, St. Louis, MO, USA, unless otherwise stated.

### Western blot to verify antibody specificity

A mouse was sacrificed by cervical dislocation and the brain was dissected. One tablet of protease inhibitor cocktail (Roche Diagnostics, Switzerland) was dissolved in 50ml PBS (137.0mM NaCl, 2.7mM KCl, 10.0mM Na_2_HPO_4_, pH 7.4) and 5 volumes were added to the sample and the brain sample was homogenized using a Dounce homogenizer. Proteins were centrifuged for 10min at 17000 rpm and the supernatant was discarded and the pellet was dissolved in 5ml PBS/inhibitor and the proteins were quickly spun. The supernatant (S_0_) was collected and the pellet was dissolved in 1ml homogenization buffer (50.0mM Tris, 150.0mM NaCl, 4.0mM MgCl, 0.5mM EDTA, 2% Triton-X and 1 protease inhibitor cocktail tablet/50ml buffer). The dissolved pellet was centrifuged at 10000 rpm for 10min and the supernatant (S_1_) was collected and the pellet (P_1_) was dissolved in 1ml homogenization buffer. The dissolved pellet (P_1_) was centrifuged again and the supernatant (S_2_) was collected and the pellet (P_2_) was dissolved in 1ml homogenization buffer. Protein concentrations were measured using the D_C_ protein assay kit (Bio-Rad, Hercules, CA, USA), following manufactures protocol, in 96 well BRANDplates® pureGrade^TM^ (BRAND GMBH, Germany). Sample preparation and electrophoresis was performed as described in [14] and 200μg of proteins from supernatant (S_2_) were loaded in the wells. Proteins were transferred to an immobilian Transfer Membrane (PVDF, 0.45μm, Millipore, Germany) using the Trans-Blot® Turbo™ Mini PVDF Transfer Packs (Bio-Rad, Hercules, CA, USA) in the Trans-Blot® Turbo™ Transfer System for 3min (Bio-Rad, Hercules, CA, USA), following manufactures protocol. The membrane was incubated in blocking buffer (5% Blotting grade blocker Non-fat dry Milk (Bio-Rad, Hercules, CA, USA) in TTBS (0.15M NaCl, 0.01M Trizma base, 0.05% Tween-20, pH = 8.0)) for 1h, before incubation in the custom made anti-SLC38A9 (Innovagen, Sweden) (NH2-MASVDGDSRHLLSEC-CONH2) diluted 1:100 in blocking buffer or in the commercially available anti-SLC38A9 (HPA043785, Sigma-Aldrich, St. Louis, MO, USA) diluted 1:100 in blocking buffer overnight at 4°C. The membrane was washed 3x10min in TTBS before 1h incubation in goat-anti-rabbit horseradish peroxidase antibody (Invitrogen, Waltham, MS, USA) diluted 1:10000 in blocking buffer. The membrane was washed 5x10min in TTBS and the blot was developed using Clarity Western ECL Substrate (Bio-Rad, Hercules, CA, USA) and visualized using a CCD camera (Bio-Rad, Hercules, CA, USA).

### Non-fluorescent DAB immunohistochemistry on free floating mouse brain sections

Sections of desired thickness (70μm) were obtained as described in [35] using a vibratome Leica VT 1200S (Leica Microsystems, Germany). Sections were rinsed in Tris-buffered saline (TBS) (0.04 M Trizma HCl, 0.01 M Trizma base, 0.15 M NaCl, pH 7.4) 4x8min before and after 40min of 0.01 M citric acid (pH 6.0) boiling at 70°C. Sections were incubated in 1% MeOH, 3% H_2_O_2_ (Merck, Germany) in TBS for 10min and rinsed in TBS again. Sections were incubated in 1% blocking reagent (Roche Diagnostics, Switzerland) for 1h followed by incubation in a custom made anti-SLC38A9 antibody (Innovagen, Sweden) diluted 1:800 in supermix (TBS, 0.25% gelatin, 0.5% Triton X-100) for 24h at 4°C. Sections were rinsed in TBS 2x1+4x8min and incubated in the secondary antibody (biotinylated goat-anti-rabbit IgG (H+L), Vector laboratories, Burlingame, CA, USA) diluted 1:400 in supermix for 1h. Sections were rinsed in TBS 5x8min before and after incubation in ABC kit (Reagent A, Reagent B (Vectastain, Vector Laboratories, Burlingame, CA, USA), diluted 1:800 in supermix for 1h. Sections were incubated in 0.05% 3.3 Diaminobenzidine tetrahydrochloride (DAB), 0.35% NiCl and 0.015% H_2_O_2_ and rinsed 4x5min in TBS and mounted on gelatinized microscope slides (Menzel Gläser, Germany). Sections were dehydrated in 70% and 95% EtOH for 5min, 100% EtOH (Solveco, Sweden) for 10min and Xylene for 20min. The DAB immunohistochemistry using the commercially anti-SLC38A9 antibody (HPA043785, Sigma-Aldrich, St. Louis, MO, USA) used in [21] was performed as stated above with the exception that the citric acid boiling and the following washes in TBS was excluded and the antibody was diluted 1:750. Sections were analyzed using a Mirax Pannoramic midi scanner with the Pannoramic Viewer software version 1.15.4 RTM (3dHistech, Hungary).

### Fluorescence immunohistochemistry on paraffin embedded mouse brain sections

Paraffin embedded sections of 7μm thickness were obtained and fluorescent immunohistochemistry was performed as described in [17], with the exceptions that anti-SLC38A9 was diluted 1:25, together with mouse anti-NeuN (1:400, Millipore, Germany), mouse anti-GAD67 (1:400, Millipore, Germany), mouse anti-GFAP (1:500, Millipore, Germany), mouse anti-glutaminase (1:100, Abcam, United Kingdom). Sections were analyzed using a fluorescence microscope (Zeiss Axioplan2 imaging) connected to a camera (AxioCam HRm) with the Axiovison 4.7 software.

### In vivo diet experiment and tissue collection

The experiment was performed in Perland *et*.*al*. 2016 [36, 37] and samples were used here to analyze the *Slc38a9* gene expression. Briefly, mice were divided in three groups (controls, starved and high-fat diet) given different diet. Group 1, control mice, were fed standard chow (R3, Lantmännen, Sweden, 5% fat), group 2, fed standard chow but starved 24h before euthanasia, and group 3, fed high-fat western diet (R638, Lantmännen, Sweden, 21% fat) for eight weeks to induce obesity. The controls and the obese mice were weighing every second week and the high-fat diet mice were significantly heavier than the controls at the day of euthanasia. Specific brain tissues (brainstem, cortex, cerebellum and hypothalamus) were dissected, and additional whole brains were dissected and cut in seven coronal regions (I-VII) using a brain matrix (Alto, 1mm, CellPoint Scientific, USA). For specific brain tissues, four mice were used in all three groups, and for larger brain regions, controls (six mice), starved (four mice), high-fat diet (six mice). RNA was extracted using Absolutely RNA Miniprep Kit (Agilent Technologies, Santa Clara, CA, USA) following the manufactures protocol. 2μg RNA was used for cDNA synthesis using High Capacity RNA-to-cDNA kit (Thermo Fisher Scientific, Waltham, MS, USA) according to manufactures protocol. cDNA from different animals, but the same brain tissue or region (I-VII) was pooled.

### Primer design and quantitative real-time PCR

Primers were designed using Beacon Design 8 (Premier Biosoft, Palo Alto, CA, USA). qPCR was performed using 5ng of pooled cDNA (5ng/μl) and the mastermix contained 2X DreamTaq Buffer (Thermo Scientific, Waltham, MS, USA), 0.2μl 25mM dNTP (Fermentas, Sweden), 0.05μl forward and reverse primer (100pmol/μl) (*Slc38a9* (F;tgctgtcattgctgtaat, R;actgagaagaaccatcct), primers for housekeeping genes *(mGapdh* (F;gccttccgtgttcctacc, R;gcctgcttcaccaccttc) *mH3a* (F;ccttgtgggtctgtttga, R;cagttggatgtccttggg) and *mActb* (F;ccttcttgggtatggaatcctgtg, R;cagcactgtgttggcatagagg)) (Invitrogen, Germany), 0.5μl 10X SYBRGreen (1:50000, diluted in TE buffer, pH 7.8, (Invitrogen, Germany)) and 0.08μl DreamTaq polymerase (5U/μl, Thermo Scientific, Waltham, MS, USA) and water was added to a total reaction volume of 20μl. All cDNA samples were run in triplicates and water was used as a negative control. The iCycler real-time detection instrument (Bio-Rad, Hercules, CA, USA) was used. Amplification was performed as follows; initial denaturation, 95.0°C for 30s, 45 cycles of: 95.0°C for 10s, 55.8°C for 30s and 72.0°C for 30s. Cycling was followed by melt curve performance for 81 cycles, starting at 55.0°C, with 10s intervals and steps of 0.5°C.

### Analysis of qPCR data

The MyIQ (Bio-Rad, Hercules, CA, USA) software was used to obtain Ct-values for all samples. The raw Ct-values were compared and outliers were removed if the difference was greater than 0.99 between triplicates. Primer efficiency was calculated with LinRegPCR software [38], after outliers were removed with Grubbs test (GraphPad software, La Jolla, CA, USA). GeNorm [39] was used to calculate if the housekeeping genes were stable. The geometric mean of the housekeeping genes was used as normalization factors and the relative mRNA expression was calculated. For the specific brain tissues, two different qPCR runs were analyzed as one set of data, i.e. hexaplicates per treatment and tissue, using the mean value of the primer efficiency from both runs (run 1, 2.0334±0.18, run 2, 2.0397±0.12) and each tissue was corrected separately. For the larger brain regions, the qPCRs were repeated twice and analyzed separately and one representative run is presented. Unpaired t-tests were performed between controls and the starved group or the high-fat diet group, using GraphPad Prism 5 (Graph Pad software, La Jolla, CA, USA), and the significance levels were Bonferroni corrected for multiple comparison, (*p≤0.049375, **p≤0.009975, ***p≤0.001).

## Results

### Verification of the antibodies used for immunostaining

**Fig 1:**
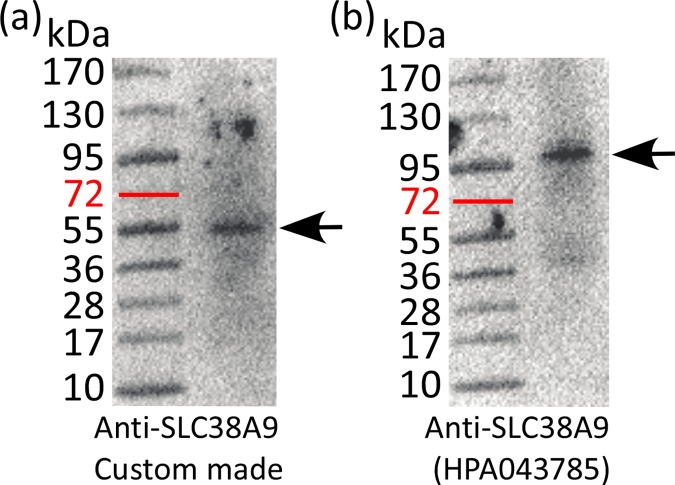
Western blot of the anti-SLC38A9 antibodies. Western blot was used to verify the antibodies. (a) The blot of the custom made anti-SLC38A9 antibody displayed a band with approximately size of 55kDa, which is ±10kDa of the predicted size of 63kDa of the SLC38A9 protein. (b) The blot of the commercial anti-SLC38A9 (HPA043785) antibody displayed a band with approximately size of 95kDa.

The specificity of the antibodies used for protein localization in mouse brain was verified using western blot on mouse brain homogenate. The predicted size of the SLC38A9 protein was found to be 63.4kDa (NP_848861). For the custom made antibody, one band was detected with approximate size of 55kDa (Fig 1A), which is in range of ±10kDa from the predicted size, and hence the blot verifies the antibody specificity. For the commercially available anti-SLC38A9 antibody, one band was detected with approximately size of 95kDA (Fig 1B).

### SLC38A9 immunostaining of the custom made antibody in cortex, thalamus, hippocampus, hypothalamus, brainstem and cerebellum

**Fig 2:**
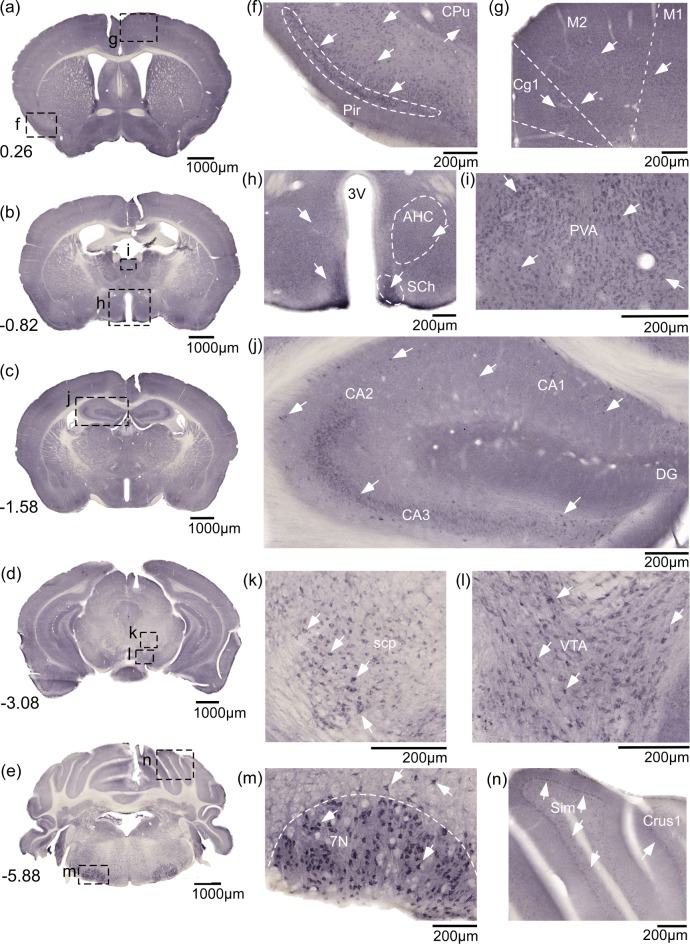
SLC38A9 immunostaining is abundant in mouse brain using a custom made anti-SLC38A9 antibody. Non-fluorescent DAB immunohistochemistry on free floating mouse brain sections, overview pictures (a-e) and close ups (f-n) with adjacent scale bars. (a) Bregma 0.26mm, (b) Bregma -0.82mm, (c) Bregma -1.58mm, (d) Bregma -3.08mm, (e) Bregma -5.88mm, (f) piriform cortex (Pir) and caudate putamen (striatum) (CPu), Bregma 0.26mm, (g) cingulate cortex, area 2 (Cg2), secondary motor cortex (M2) and primary motor cortex (M1), Bregma 0.26mm, (h) Third ventricle (3V), Suprachiasmatic nucleus (SCh) and anterior hy area, central (AHC), Bregma -0.82mm, (i) paraventricular thalamic nucleus anterior, (PVA), Bregma -0.82mm, (j) hippocampus, field CA1 hippocampus (CA1), field CA2 hippocampus (CA2), field CA3 hippocampus (CA3) and dentate gyrus (DG), Bregma -1.58mm, (k) superior Cb peduncle (scp), Bregma -3.08mm, (l) ventral tegmental area (VTA), Bregma -3.08mm, (m) Facial nucleus (7N), Bregma -5.88mm, (n) Purkinje cell layer in simple lobule (Sim) crus 1 ansiform lobule (Crus1), Bregma -5.88mm.

Non-fluorescent immunohistochemistry on 70μm free floating mouse brain sections was performed using the custom made anti-SLC38A9 antibody (Fig 2A–2E overview pictures and 2F–2N close ups), to provide a comprehensive profile of the protein expression in mouse brain. Immunoreactivity was detected throughout the mouse brain. In cortex the staining was especially prominent in piriform cortex (Pir) (Fig 2F) and evenly distributed in cortex layer 2–6, in cingulate cortex, area 1(Cg1), primary motor cortex (M1) and secondary motor cortex (M2) (Fig 2G). Staining was detected in hypothalamic areas around the third ventricle (3V), in suprachiasmatic nucleus (SCh) and in anterior hypothalamic area, central (AHC) (Fig 2H). Immunoreactivity was found in thalamic parts, e.g. paraventricular thalamic nucleus (PVA) (Fig 2I). Moreover, SLC38A9 staining was found in hippocampus, in field CA3 the staining was more intense while only specific cells were stained in field CA1 and CA2 and dentate gyrus (DG) (Fig 2J). Cells were also stained in superior Cb peduncle (scp) (Fig 2K) and in the ventral tegmental area (VTA) (Fig 2L). In the posterior parts of the brain, SLC38A9 immunoreactivity was detected in brainstem, with large cells stained in the area facial nucleus (7N) (Fig 2M), and in the Purkinje cell layer in cerebellum (Fig 2N). The described brain areas were identified using, The Mouse Brain, by Franklin and Paxinos 2007.

### SLC38A9 immunostaining of the commercially available antibody in cortex, thalamus, hippocampus, hypothalamus, brainstem and cerebellum

**Fig 3:**
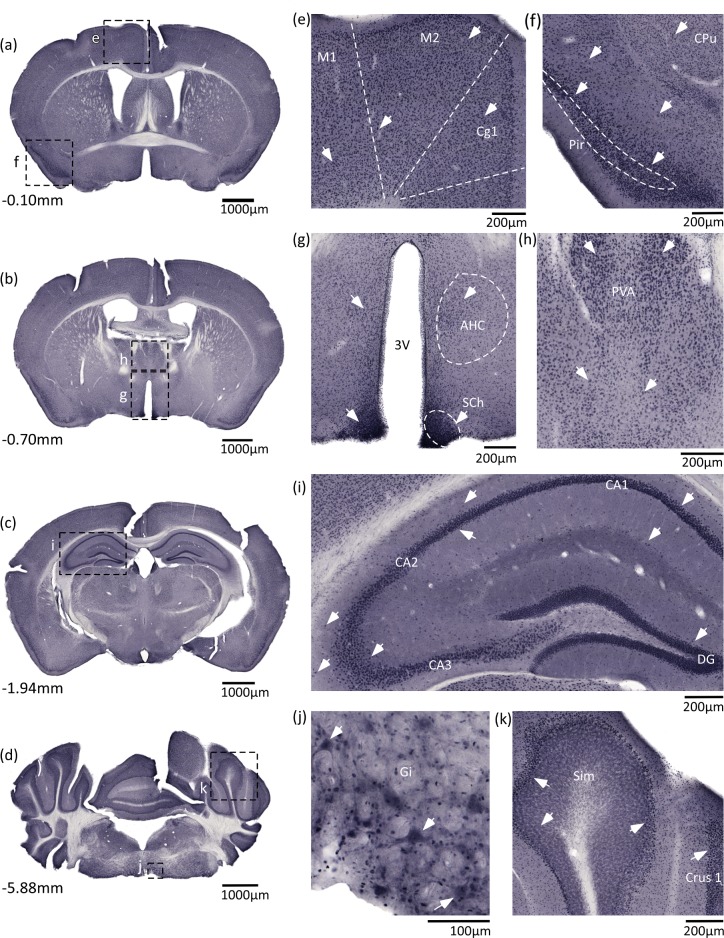
SLC38A9 immunostaining is abundant in mouse brain using a commercial anti-SLC38A9 antibody. Additional DAB immunohistochemistry on free floating mouse brain sections was performed using a commercially available anti-SLC38A9 antibody, to verify the staining of the custom made anti-SLC38A9 antibody. Overview pictures (a-d) and close up pictures (e-k) with adjacent scale bars. (a) Bregma -0.10mm, (b) Bregma -0.70mm, (c) Bregma -1.94mm, (d) Bregma -5.88mm, (e) cingulate cortex, area 1 (Cg1), primary motor cortex (M1) and secondary motor cortex (M2), Bregma -0.10mm, (f) piriform cortex (Pir) and caudate putamen (striatum) (CPu), Bregma -0.10mm, (g) third ventricle (3V), suprachiasmatic nucleus (SCh) and anterior hypothalamic area, central (AHC), Bregma -0.70mm, (h) paraventricular thalamic nucleus (PVA), Bregma -0.70mm, (i) hippocampus, field CA1 hippocampus (CA1), field CA2 hippocampus (CA2), field CA3 hippocampus (CA3) and dentate gyrus (DG), Bregma -1.94mm, (j) gigantocellular reticular nucleus (Gi), Bregma -5.88mm, (k) Purkinje cell layer in simple lobule (Sim) and crus 1 ansiform lobule (Crus1), Bregma -5.88mm.

To verify the staining of the custom made anti-SLC38A9 antibody further, a complementary non-fluorescent immunohistochemistry was run using the commercially anti-SLC38A9 antibody (Fig 3A–3D overview pictures and 3E–3K close ups). SLC38A9 staining was found in cortex, with evenly distributed staining in cortex layer 2–6, in cingulate cortex, area 1 (Cg1), primary motor cortex (M1) and secondary motor cortex (M2) (Fig 3E), with high intensity in piriform cortex (Pir) (Fig 3F). Immunoreactivity was found in hypothalamic areas around third ventricle (3V), in suprachiasmatic nucleus (SCh) and anterior hypothalamic area, central (AHC) (Fig 3G). In addition, staining was also found in thalamic areas, paraventricular thalamic nucleus (PVA) (Fig 3H). Evenly distributed staining was found in hippocampus, in dentate gyrus (DG) and in field CA1, CA2 and CA3 (Fig 3I). In brainstem, large cells were stained in gigantocellular reticular nucleus (Gi) (Fig 3J) and in cerebellum intense immunoreactivity was found in the Purkinje cell layer (Fig 3K).

### SLC38A9 staining in inhibitory and excitatory neurons

**Fig 4:**
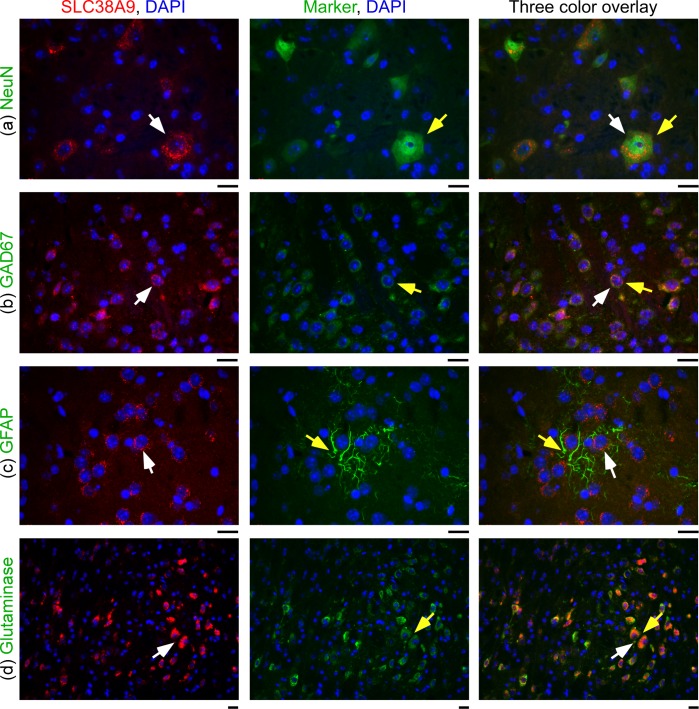
SLC38A9 staining co-localize with GABAergic and glutamatergic neurons. Fluorescence immunohistochemistry (a-d) on mouse brain paraffin embedded sections with SLC38A9 immunostaining in red, protein markers in green and the nuclear marker DAPI in blue. All scale bars are 20μm. White arrows indicate cells with SLC38A9 immunoreactivity and yellow arrows indicate cells with immunoreactivity of the markers. (a) The neuronal marker NeuN co-localizes in the 10th cerebellar lobule (10cb), Bregma -6.72mm. (b) The GABAergic neuronal marker GAD67 co-localized with SLC38A9 in cells close to third ventricle (3V), in anterior hypothalamic area, post (AHP), Bregma -1.22mm. (c) SLC38A9 and the astrocyte marker GFAP do not overlap in the area around third ventricle (3V), reuniens thalamic nucleus (Re), Bregma -0.70mm. (d) The enzyme glutaminase is expressed in glutamatergic neurons and overlap with SLC38A9 in the area B9 serotonin cells (B9), Bregma -4.36mm.

Fluorescent immunohistochemistry was used to determine the co-localization of the custom made anti-SLC38A9 and specific cell makers (Fig 4). The antibody was hybridized together with specific cell markers on 7μm paraffin embedded mouse brain sections. SLC38A9 immunofluorescence co-localized with the neuronal marker NeuN in 10th cerebellar lobule (10Cb) at Bregma -6.72mm (Fig 4A), and with the GABAergic neuronal marker GAD67 in cells in the area anterior hypothalamic area, post (AHP) at Bregma -1.22mm (Fig 4B). No co-expression with the astrocytic marker GFAP was observed in reuniens thalamic nucleus (Re) at Bregma -0.70mm, (Fig 4C). Furthermore, SLC38A9 immunoreactivity and the enzyme glutaminase co-localized in glutamatergic neurons in the area B9 serotonin cells (B9) at Bregma -4.36mm, (Fig 4D).

### *Slc38a9* changes in mouse brain following starvation and high-fat diet

**Fig 5:**
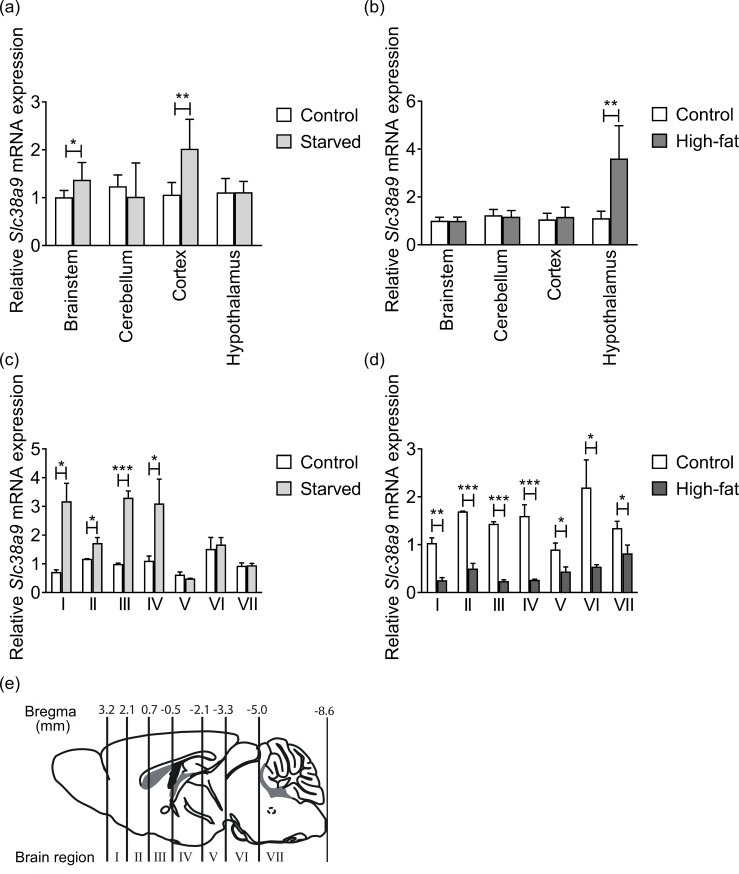
*Slc38a9* gene expression in mouse brain following starvation and high-fat diet. Relative *Slc38a9* mRNA expression ±SD are plotted and compared with controls (Unpaired t-tests, significance adjusted for multiple testing, *p≤0.049375, **p≤0.009975, ***p≤0.001, n represents technical replicates). (a) Control group (white bars), Starved group (light grey bars), n = 6 for both groups and all tissue tested, except n = 5 for starved group in cerebellum, Brainstem (p = 0.0442), Cerebellum (p = 0.4865), Cortex (p = 0.0055), Hypothalamus (p = 0.9803). (b) Control group (white bars), High fat diet group (dark grey bars), n = 6 for both groups and all tissue tested, Brainstem (p = 0.9111), Cerebellum (p = 0.6745), Cortex (p = 0.5967), Hypothalamus (p = 0.0014). (c) Control group (white bars, (region I, II, III and VI, n = 2, region IV, V and VII, n = 3), Starved group (light grey bars, (n = 3 for all regions except for regions I and VII were n = 2)). (I (p = 0.0310), II (p = 0.0297), III (p = 0.0009), IV (p = 0.0159), V (p = 0.0743), VI (p = 0.6162) and VII (p = 0.8486)). (d) Control group (white bars, (region I, II, III and VI, n = 2, region IV, V and VII, n = 3)), High-fat diet group (dark grey bars, (n = 3 for all brain regions except for region V, n = 2)). (p = 0.0016), II (p = 0.0007), III (p<0.0001), IV (p = 0.0006), V (p = 0.0266), VI (p = 0.0124) and VII (p = 0.0151). (e) Representation of the mouse brain displaying the brain regions I-VII, with corresponding Bregma numbers (mm).

To study the *Slc38a9* gene expression changes *in vivo* in response to nutrient availability, mice were starved for 24h or fed with high-fat diet for 8 weeks before euthanasia. Specific brain tissues together with larger brain regions (I-VII) were dissected for analysis (Fig 5). Following 24h of starvation, the *Slc38a9* gene expression was significantly upregulated in brainstem (p = 0.0442) and cortex (p = 0.0055) compared with controls, while no significant regulation was found in cerebellum (p = 0.4865) or hypothalamus (p = 0.9803) (Fig 5A). After high-fat diet, *Slc38a9* was upregulated in hypothalamus (p = 0.0014) (Fig 5B), but not altered in brainstem (p = 0.9111), cerebellum (p = 0.6745) or cortex (p = 0.5967). Furthermore, *Slc38a9* was significantly upregulated in the starved group in brain regions; I (p = 0.0310), II (p = 0.0297), III (p = 0.0009) and IV (p = 0.0159), while expression was unaffected in brain regions; V (p = 0.0743), VI (p = 0.6162) and VII (p = 0.8486) (Fig 5C). The *Slc38a9* gene expression was significantly downregulated in all brain regions from the obese mice, I (p = 0.0016), II (p = 0.0007), III (p<0.0001), IV (p = 0.0006), V (p = 0.0266), VI (p = 0.0124) and VII (p = 0.0151) (Fig 5D). Fig 5E displays a schematic picture of the mouse brain divided in seven regions (I-VII) with the corresponding Bregma numbers (mm), image was adopted from [36].

## Discussion

SLC38A9 is characterized as a lysosomal component of the amino acid sensing Ragulator-RAG GTPase complex controlling the mechanistic target of rapamycin complex 1 (mTORC1) [20–22], one of the major amino sensing pathways in mammalian cells. Here we have characterized the protein expression of SLC38A9 in the mouse brain. Furthermore, due to the established role of SLC38A9 in the mTORC1 pathway, we found it interesting to study the *Slc38a9* gene expression alterations following *in vivo* starvation and high-fat diet. *Slc38a9* expression was analyzed in specific brain tissues associated with feeding behavior, and in larger brain regions, since neuronal circuits can project beyond specific brain structures, to provide a more comprehensive understanding of how altered energy intake can affect the overall gene expression in the mouse brain.

Abundant SLC38A9 immunoreactivity was found throughout the mouse brain, in cortex, thalamus, hippocampus, hypothalamus, brainstem and cerebellum, and two antibodies were used to verify the staining. The staining of both antibodies was similar, although the staining from the commercial antibody was more intense. However, the staining of the commercially available anti-SLC38A9 was broader in hippocampus and stained all fields as well as dentate gyrus, while the custom made antibody stained some cells in dente gyrus and field CA1 and CA2, while abundant staining was only found in field CA3. Previously characterized members from the SLC38 family are in brain, either located to neurons or astrocytes or both. SLC38A9 immunoreactivity was located to GABAergic and glutamatergic neurons, while no staining was found in astrocytes. This is similar to what has been found for SLC38A6 [33], SLC38A7 [17] and SLC38A8 [14].

CNS control eating behavior, and receive signals about energy status via various neuronal circuits [40]. Hypothalamus is one of the key structures associated with regulation of metabolism and mTORC1 activity [41]. In hypothalamus, the arcuate nucleus (Arc), close to the third ventricle, integrate signals about energy status and further control metabolism and energy balance via mTORC1 [42]. SLC38A9 immunostaining was found in hypothalamic areas around the third ventricle (3V), which was not surprising, considering the function of SLC38A9 as a signal integrator about amino acid levels. In hypothalamus, the mTORC1 pathway is activated in response to amino acids, glucose and growth factors, resulting in reduced food intake [43]. However, during diet induced obesity, the mTORC1 pathway is downregulated in hypothalamus [44]. The gene expression of *Slc38a9* was found to be un-altered following starvation, but upregulated after high-fat diet in hypothalamus. The diet induced obesity paradigm is a long term treatment while the starvation is short term. This could be the reason why the high-fat diet influences *Slc38a9* gene expression while the starvation paradigm does not. It is possible that the upregulation of *Slc38a9* in hypothalamus is to enable mTORC1 activation and compensate for the diet-induced loss of mTORC1 activity.

In the mammalian brain, the piriform cortex is shown to sense uncharged tRNAs and inadequate levels of amino acids in food. The cells in anterior piriform cortex are sensitive to amino acid deficiency and the eukaryotic initiation factor 2 (eIF2) is phosphorylated by the GCN2 kinases in the absence of amino acids [45]. In the amino acid responsive (AAR) pathway, the detection of amino acid limitation begins with the sensing of uncharged tRNA, which accumulates during starvation, by the GCN2 kinases, which results in phosphorylation of the eukaryotic initiation factor 2α (eIF2α) [46, 47]. The translation is then strongly inhibited and the activating transcription factor 4 (ATF4) is therefore transcriptionally upregulated, leading to upregulation of genes holding amino acid responsive elements (AARE). The overall effect of this pathway is decreased protein synthesis [48]. The importance of GCN2 and eIF2α in response to limited levels of amino acids has been confirmed in mouse models [49, 50] and this pathway seems to be important in CNS to identify variations in amino acid composition in food [45, 51, 52]. Several amino acid transporters from the SLC superfamily e.g. SLC7A1 [53], SLC7A5 [54], SLC7A11 [55], SLC1A4 [56], SLC1A5 [57], SLC3A2 [57] and also the SLC38 family member, SLC38A2 [58, 59], are transcriptionally upregulated during amino acid starvation and these genes also have identified responsive elements. In addition, SLC1A5 [60], SLC7A5/SLC3A2 [60] and SLC38A2 [61–63] have also been found to be involved in the activation of mTORC1. Intense SLC38A9 immunostaining was found in piriform cortex suggesting a potential role in sensing inadequate amino acid levels. In addition, the upregulation of *Slc38a9* in cortex following starvation, suggest that *Slc38a9* could be under control of responsive elements and respond to amino acid deprivation in a similar way as other SLCs.

In previous studies in cells overexpressing SLC38A9, mTORC1 activation was shown to be insensitive to amino acid deprivation, and loss of SLC38A9 reduced mTORC1 activation by amino acids [21, 22]. Furthermore, mTORC1 activity was also inhibited when SLC38A9 was depleted in the presence of amino acids, as well as after amino acid supplementation following deprivation [20]. Amino acids increase the mTORC1 activation on lysosomes and can thereby control metabolism and cell growth [26]. Following starvation, *Slc38a9* was significantly upregulated in brain regions I-IV (Bregma 3.2 to -2.1mm), and specifically in cortex and brainstem. Brain regions from section I-IV all comprise tissue from cortex among others. However, in brain region VII, encompassing brainstem, no alteration was found. The signal from brainstem could have been diluted in the larger sample as this region also include cerebellum, which was not altered. Following high-fat diet, *Slc38a9* was downregulated in all brain regions, I-VII (Bregma 3.2 to -8.6mm). Furthermore, no downregulation of *Slc38a9* was detected in brainstem, cerebellum or cortex, but instead upregulation in hypothalamus was found. The upregulation in hypothalamus could be compensatory for the loss of mTORC1 activity induced by obesity, as earlier discussed. In the larger brain regions, the effects on gene expression is a sum of the expression in all structures within that region, and signals could hence be increased or decreased, which could explain the differences found in specific tissues and brain regions. In our feeding experiments, the overall gene expression of *Slc38a9* was altered in the opposite direction depending on nutritional availability. During amino acid starvation, cells are exposed to low levels of amino acids and to maintain protein synthesis and cellular growth the mTORC1 pathway must be activated. It is possible that the requirement of SLC38A9 in order to sense amino acids increases, without this sensing of amino acid the mTORC1 pathway remain inactive. The increased need for SLC38A9 could explain the upregulation of *Slc38a9* following starvation. After high-fat diet, the cells have plenty of amino acids, growth factors and nutrients capable for mTORC1 activation, and the need for SLC38A9 protein translation might be decreased, and hence *Slc38a9* is downregulated.

In conclusion, SLC38A9 immunostaining was found throughout the mouse brain in cortex, hypothalamus, thalamus, hippocampus, brainstem and the Purkinje cell layer in cerebellum. More specifically, we found staining in hypothalamic areas and piriform cortex, areas known to be involved in amino acid sensing and signaling. SLC38A9 immunofluorescence co-localized with markers for both GABAergic and glutamatergic neurons. *Slc38a9* gene expression was altered in mouse brain following *in vivo* starvation and high-fat diet. Following starvation, upregulation of *Slc38a9* was found specifically in brainstem and cortex, and in brain regions between Bregma 3.2 to -2.1mm. After high-fat diet, upregulation of *Slc38a9* was specifically found in hypothalamus, while *Slc38a9* was downregulated throughout the brain between Bregma 3.2 to -8.6mm.

## Abbreviations

SLCs: Solute carriers
SNAT: Sodium-coupled neutral amino acid transporter
mTORC1: mechanistic target of rapamycin complex 1
AAR: amino acid responsive
AARE: amino acid responsive elements
